# A Prospective Self-Controlled Study of Indocyanine Green, Radioisotope, and Methylene Blue for Combined Imaging of Axillary Sentinel Lymph Nodes in Breast Cancer

**DOI:** 10.3389/fonc.2022.803804

**Published:** 2022-02-09

**Authors:** Yuting Jin, Long Yuan, Yi Zhang, Peng Tang, Ying Yang, Linjun Fan, Li Chen, Xiaowei Qi, Jun Jiang

**Affiliations:** Department of Breast Surgery, Southwest Hospital, Third Military Medical University (Army Medical University), Chongqing, China

**Keywords:** breast cancer, indocyanine green, radioisotope, combined imaging, sentinel lymph node biopsy

## Abstract

**Purpose:**

This self-controlled study aimed to clarify whether indocyanine green (ICG) could be an alternative tracer in the absence of radioisotope (RI) for combined imaging of axillary sentinel lymph node (SLN) in breast cancer.

**Methods:**

Primary breast cancer, clinically axillary node-negative patients (n = 182) were prospectively enrolled from March 2015 to November 2020. ICG, methylene blue (MB), and RI were used to perform axillary sentinel lymph node biopsy (SLNB). The main observation index was the positivity of ICG + MB vs. RI + MB in axillary SLNB; the secondary observation indicators were the axillary SLN detection rate, mean number of axillary SLNs detected, mean number of metastatic axillary SLNs detected, and safety.

**Results:**

All 182 patients had axillary SLNs; a total of 925 axillary SLNs were detected. Pathological examination confirmed metastatic axillary SLN in 42 patients (total of 79 metastatic SLNs). Positivity, detection rate of SLNs, detection rate of metastatic SLNs, and the number of metastatic SLNs detected were comparable with RI+MB and ICG+MB (*p* > 0.05). The mean number of axillary SLNs detected was significantly higher with ICG+MB than with RI+MB (4.99 ± 2.42 vs. 4.02 ± 2.33, *p <* 0.001). No tracer-related adverse events occurred.

**Conclusions:**

ICG appears to be a safe and effective axillary SLN tracer, and a feasible alternative to RI in combined imaging for axillary SLN of breast cancer.

## Introduction

Sentinel lymph node biopsy (SLNB) for breast cancer is a minimally invasive technique that can provide accurate axillary staging (1, 2). When SLNB is negative, axillary lymph node dissection (ALND) (1, 3, 4) can be avoided, and the patient is spared the suffering caused by complications such as upper limb lymph edema, nerve damage, local pain, numbness, and shoulder stiffness (1). A key factor for the accuracy of axillary SLNB is the tracer that is used (5). Currently, the commonly used tracers are radioisotope (RI), blue dye, and RI plus blue dye (dual-tracer method). The American Society of Clinical Oncology (ASCO) recommends the dual tracer method for axillary SLNB because of its high detection rate (>90%), low false-negative rate (5%–10%), and the short learning curve (6). However, high cost, radiation exposure, relatively complicated surgical preparations, painful preoperative injections, and the need for nuclear medicine personnel are some of the disadvantages associated with RI use (7). In addition, the RI method cannot provide clear and intuitive visual guidance during the surgery and may therefore impair the surgeon’s ability to locate the sentinel lymph node. With the increasing demand for day surgery, the relatively long time taken for the application of RI tracing cannot be ignored either (8). Therefore, there is much ongoing research to identify lymphatic tracers that could replace RI.

In recent years, indocyanine green (ICG), a common near-infrared fluorescent tracer, has been increasingly used in axillary SLNB; the advantages are lack of radiation exposure, ease of use, and a short learning curve. The passage of ICG through the lymphatic vessels can be visualized in real time, greatly facilitating surgery and shortening operation time. There is also no “blue tattooing” or blue dye pollution in the operation area (9, 10). The success rate with ICG has been found to be higher than that with blue dye and comparable to that with RI (11). In a previous prospective controlled study, we obtained comparable results with the combination of ICG plus methylene blue (MB) and RI plus MB (12). However, some authors have found that body mass index is significantly related to the detection of ICG fluorescence in skin lymphatic vessels, and age and obesity may reduce the probability of successful axillary SLNB (3, 13–16).

This prospective study aimed to clarify whether ICG could be an alternative tracer in the absence of RI for axillary SLN mapping in breast cancer. We compared the efficacies of ICG+MB with RI+MB for detection of axillary SLN in early breast cancer patients; to avoid the effects of BMI, age, and anatomical variations of the lymphatic system, we adopted a self-controlled protocol.

## Patients and Methods

### Patients

**Figure 1:**
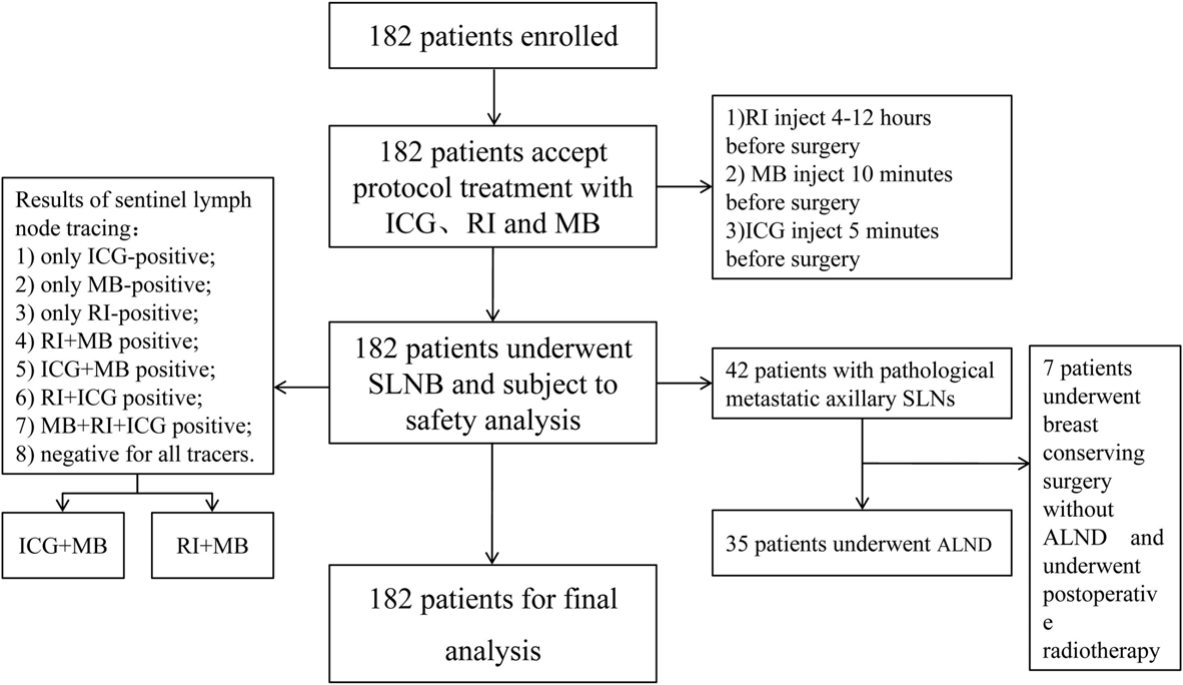
Flow diagram.

Between March 2015 and November 2020, a total of 182 patients with primary breast cancer scheduled to undergo axillary SLNB were enrolled in this study. All patients had diagnosis confirmed by core needle biopsy and were clinically and radiologically lymph node negative. Patients with clinically or radiologically suspicious lymph nodes, inflammatory breast cancer, distant metastases, previous axillary surgery, or hypersensitivity to iodine or ICG were excluded from the study (**Figure 1**).

This study was approved by the Medical Ethics Committee of the Southwest Hospital, Third Military Medical University (Army Medical University), Chongqing, China (clinical trial registration no. ChiCTR2000030729). All patients signed informed consent forms before the operation. This study strictly followed the Declaration of Helsinki and relevant clinical trial specifications, laws, and regulations.

### Reagents and Equipment

The tracers used in this study were 1% MB solution (Jumpcan Pharmaceutical Group Co., Ltd., Taixing, China); 99Tcm-colloids (3.7×10^7^ Bq, Shihong Pharmaceutical, Beijing, China); and 1.25% ICG solution (Dandong Pharmaceutical Co., Ltd., Liaoning, China). The fluorescent vascular imaging system (MDM-I, Mingde, Langfang, China) and Neo2000 Gamma Detection System (Neoprobe Corporation, OH, USA) were used to detect the signal of ICG-positive and RI-positive lymph nodes.

### Procedure

**Figure 2:**
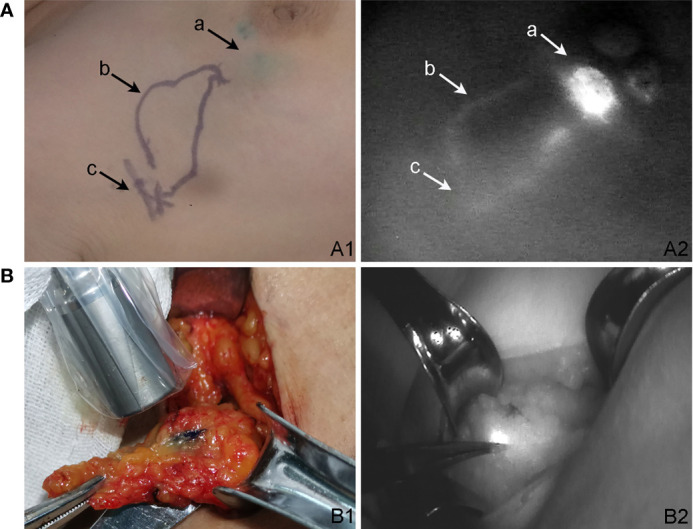
Detection of tracer-positive lymph node. **(A)** ICG skin development. **(A1)** ICG percutaneous lymphography and sentinel lymph node localization. **(A2)** Lymphatic pathway under the fluorescent detector. (a) ICG injection location. (b) Lymphatic vessels highlighted by ICG. (c) Sentinel lymph node location. **(B)** The development of sentinel lymph nodes after subcutaneous incision. **(B1)** Blue-stained lymph node was detected by the γ detector. **(B2)** The same lymph node was ICG positive under the fluorescent detector."

This study was a self-controlled trial. Confounding factors such as BMI, age, and individual differences in lymphatic anatomy were reduced by the use of three different tracers, indocyanine green, radioisotope, and methylene blue, in the same patient. The tracers were injected into the subareolar area; 1 ml of RI was injected subdermally 4–12 h before surgery, 1 ml of 1% MB was injected subdermally in the disinfected periareolar region 10 min before surgery, and 1 ml of 1.25% ICG was injected intradermally 5 min before surgery, followed by massage for another 5 min (12). Lymph nodes were detected by gamma detector, naked eyes and fluorescent detector, respectively (**Figure 2**). The lights of the operating area should be turned off when detecting lymphatic and lymph nodes by a fluorescent detector. Axillary SLNB was performed by experienced surgeons following standard operating procedures. Patients with negative axillary SLN on intraoperative frozen section examination did not undergo ALND. For patients with definite axillary SLN metastasis or indeterminate results, the decision on whether to proceed with ALND was according to the current guidelines and the patient’s preoperatively expressed preference.

MB-positive lymph nodes were those that appeared blue to the naked eye or those with blue-stained lymph vessels entering them. RI-positive lymph nodes were those in which the gamma detector showed threshold count >10% of the maximum lymph node count (17). ICG-positive lymph nodes were those that showed fluorescent bright spots on the fluorescence imager. All tracer-positive lymph nodes were removed during the resection; other suspicious lymph nodes (large, hard) were also removed. The “tracer status” of the removed lymph nodes was recorded before they were sent for intraoperative frozen section examination. There were eight possible tracer combinations for each lymph node: 1) only ICG-positive; 2) only MB-positive; 3) only RI-positive; 4) RI+MB positive; 5) ICG+MB positive; 6) RI+ICG positive; 7) MB+RI+ICG positive; or 8) negative for all tracers.

For the purpose of this analysis, the total number of axillary SLNs detected was defined as the tracer-positive lymph nodes (18).

### Pathological Examination and Postoperative Treatment

Isolated tumor cells (ITCs) refer to tumor lesions in lymph nodes with a diameter less than 0.2 mm or tumor cells in a single section with a diameter less than 0.2 mm. Micrometastasis was defined as the tumor metastasis with the largest diameter more than 0.2 mm but no more than 2 mm. Metastatic lymph nodes with a maximum diameter of tumor metastasis more than 2 mm were considered to be macrometastases. For patients who did not receive neoadjuvant chemotherapy, metastatic axillary SLNs were those with macrometastasis and micrometastasis; ITCs and no metastasis were defined as axillary SLN negative. For patients who received neoadjuvant chemotherapy, metastatic axillary SLN were those with axillary SLN macrometastasis, micrometastasis, and ITCs; no metastasis was defined as negative. Diagnosis and treatment were according to National Comprehensive Cancer Network (NCCN) guidelines and the Chinese Anti-Cancer Association guidelines for breast cancer.

Tracer-related complications occurring within 1 week of injection of the tracer were recorded; the complications included regional or systemic allergic reactions, infection at the injection site, and serious adverse events requiring clinical treatment or causing disability or death.

### Statistical Analysis

Positivity was defined as the number of patients with metastatic SLN detected by a tracer or combination divided by the total number of patients with metastatic SLN. Axillary SLN detection rate was defined as the number of patients with SLNs detected by one or more of the tracers divided by the total number of patients. Patients with negative axillary SLN did not undergo ALND surgery, and so the true positive rate, true negative rate, positive predictive value, and negative predictive value could not be calculated (8). The false-negative rate is difficult to calculate either. The main observation index was positivity. The secondary observation indicators were the axillary SLN detection rate, the mean number of axillary SLNs detected, the mean number of metastatic axillary SLNs detected, and safety.

The paired chi-square test was used to compare positivity, total axillary SLN detection rate, and metastatic axillary SLN detection rate. The paired t test was used to compare the number of axillary SLNs detected and the number of metastatic axillary SLNs detected. Average values are presented as mean ± SD. Because of the multiple comparisons involved, corrected statistical significance was set at p < 0.002 (α/number of comparisons, α = 0.05). SPSS 25.0 (IBM Corp., Armonk, NY, USA) and Microsoft Office Excel 2007 were used for statistical analysis.

## Results

### Demographics and Tumor Characteristics

A total of 182 patients (median age, 48 years; age range, 31–74 years) were enrolled in this study. The mean BMI was 23.5 kg/m^2^ (range, 18.4–42.4 kg/m^2^). The tumor was located in the upper outer quadrant in 38.6% of patients. Neoadjuvant chemotherapy was administered to 30 (16.5%) patients. 42 patients (24.2%) had metastatic axillary SLNs, ALND was performed for 35 (19.2%) patients, and 7 patients with metastatic axillary SLNs who underwent breast conserving surgery did not receive ALND according to the results of the Z0011 study (19). No patient underwent a second operation due to inconsistent pathological examinations. **Table 1** summarizes the clinicopathological characteristics of the patients.

**Table 1:** Demographic and clinical characteristics of the 182 patients.

Characteristic		n	%
Age (years)	<50	104	57.1
	≥50	78	42.9
Menopausal status	Premenopausal	109	59.9
	Postmenopausal	73	40.1
BMI^a^ (kg/m^2^)	<24	106	58.2
	≥24	76	41.8
Tumor side	Left	100	54.9
	Right	82	45.1
Tumor location	Upper outer quadrant	66	36.3
	Lower outer quadrant	30	16.5
	Upper inner quadrant	48	26.4
	Lower inner quadrant	17	9.3
	Nipple–areolar area	21	11.5
Neoadjuvant chemotherapy	Yes	30	16.5
	No	152	83.5
Histological type	*In situ*	13	7.1
	Invasive non-specific cancer	153	84.1
	Invasive specific cancer	16	8.8
T stage	Tis^b^	13	7.1
	T1	90	49.5
	T2	78	42.9
	T3	1	0.5
Type	Luminal A	54	29.7
	Luminal B	47	25.8
	Her-2 positive	57	31.3
	TNBC^c^	24	13.2
Breast surgery	Mastectomy	130	71.4
	Lumpectomy	52	28.6
Axillary surgery	SLNB^d^	147	80.8
	SLNB+ALND^e^	35	19.2

a Body mass index.

b Tumor in situ.

c Triple-negative breast cancer.

d Sentinel lymph node biopsy.

e Axillary lymph node dissection.

### Sentinel Lymph Node Tracer Status

All 182 patients had axillary SLNs. A total of 925 axillary SLNs were detected (mean, 5.1 per patient). In two patients, the detected SLNs were only ICG positive; in both cases, pathology showed lymph node metastases. In 178 patients, there was obvious percutaneous lymphography and the SLNs were ICG positive; thus, the detection rate with ICG alone was 97.8% (178/182). The detection rate with RI+MB was 98.8% (180/182), while the detection rate with ICG+MB was 100% (182/182; **Table 2**).

**Table 2:** Axillary sentinel lymph node tracer status.

Category	Positivity^d^	SLN Detection Rate	SLN Number	Metastatic SLN Number
ICG^a^	90.5% (38/42)	97.8% (178/182)	4.63 ± 2.51	0.37 ± 0.98
MB^b^	83.3% (35/42)	89.6% (163/182)	3.12 ± 2.48	0.29 ± 0.76
RI^c^	90.5% (38/42)	94.5% (172/182)	3.30 ± 2.10	0.30 ± 0.72
RI+MB	92.9% (39/42)	98.9% (180/182)	4.02 ± 2.34	0.37 ± 0.88
ICG+MB	95.2% (40/42)	100% (182/182)	4.99 ± 2.42	0.41 ± 1.01
RI+ICG	100% (42/42)	100% (182/182)	4.93 ± 2.41	0.41 ± 1.01
ICG+MB+RI	100% (42/42)	100% (182/182)	5.08 ± 2.41	0.43 ± 1.04

a Indocyanine green.

b Methylene blue.

c Radioisotope.

d Positivity was defined as the number of patients with metastatic SLNs detected by one or more of the tracers divided by the total number of patients with metastatic SLN.

Pathological examination confirmed metastatic SLNs in 42 patients (a total of 79 metastatic axillary SLNs). In 32 patients, the metastatic nodes were successfully detected by ICG, MB, and RI. The positivity was 90.5% (38/42) for both ICG and RI, and 83.3% (35/42) for MB.

### ICG+MB vs. RI+MB

Among 42 patients with metastatic axillary SLNs, 37 patients were detected by both ICG+MB and RI+MB, 3 patients were detected only by ICG+MB, and 2 patients were detected only by RI+MB. RI+MB detected 68 metastatic SLNs in 39 patients, and ICG+MB detected 74 metastatic SLNs in 40 patients; the proportion of patients with SLN metastases identified by the two methods was not significantly different (92.9% (39/42) vs. 95.2% (40/42), *p* = 1.000; **Table 3**), and the mean number of metastatic SLNs detected by the two methods was not significantly different (0.37 ± 0.88 vs. 0.41 ± 1.01, *p* = 0.332). The overall detection rate was not significantly different with the two methods: 100% (182/182) with ICG+MB vs. 98.9% (178/182) with RI+MB. The mean number of axillary SLNs detected was higher with ICG+MB than with RI+MB (4.99 ± 2.42 vs. 4.02 ± 2.34, *p <* 0.001).

**Table 3:** Comparison of efficacies of ICG+MB and RI+MB.

Efficacy Parameter	ICG^a^+MB^b^	RI^c^+MB	*p*
Positivity^d^	95.2% (40/42)	92.9% (39/42)	1.000
SLN detection rate	100% (182/182)	98.9% (180/182)	0.480
Metastatic SLN number	0.41 ± 1.01	0.37 ± 0.88	0.332
SLN number	4.99 ± 2.42	4.02 ± 2.34	0.000

a Indocyanine green.

b Methylene blue.

c Radioisotope.

d Positivity was defined as the number of patients with metastatic SLNs detected by one or more of the tracers divided by the total number of patients with metastatic SLNs.

The metastatic SLN detection rate based on SLNs with ICG+MB was no less than that of RI+MB (93.7% (74/79) vs. 86.1% (68/79), *p* = 0.114). ICG+MB detected a total of 909 axillary SLNs, and RI+MB detected a total of 732 axillary SLNs. Therefore, the SLN detection rate based on SLNs with ICG+MB was higher than that of RI+MB (98.3% (909/925) vs. 79.1% (732/925), *p* = 0.000).

In addition, we performed a subgroup analysis. Thirty neoadjuvant chemotherapy patients and 152 non-neoadjuvant chemotherapy patients were analyzed and compared in terms of positivity, SLN detection rate, SLN number, and metastatic SLN number. The results showed that the positivity and SLN detection rates of ICG+MB and RI+MB were equal (100%) in 30 patients after neoadjuvant chemotherapy. Similarly, the positivity and SLN detection rates of ICG+MB and RI+MB were 100% in 30 neoadjuvant chemotherapy patients compared with 152 non-neoadjuvant chemotherapy patients. The SLN number and metastatic SLN number in the neoadjuvant chemotherapy group were smaller than those in the non-neoadjuvant chemotherapy group (4.37 ± 2.47 vs. 5.22 ± 2.38, *p* = 0.075; 0.23 ± 0.57 vs. 0.47 ± 1.10, *p* = 0.247). SLN number with the RI+MB method in the neoadjuvant chemotherapy group was smaller than that in the non-neoadjuvant chemotherapy group (3.07 ± 1.96 vs. 4.21 ± 2.36, *p* = 0.014). However, SLN number with the ICG+MB method showed no significant difference between the neoadjuvant chemotherapy group and the non-neoadjuvant chemotherapy group (4.30 ± 2.45 vs. 5.13 ± 2.40, *p* = 0.086). For neoadjuvant chemotherapy patients, SLN number with the ICG+MB method was greater than that with the RI+MB method (4.30 ± 2.45 vs. 3.07 ± 1.96, *p* = 0.036).

### Safety

No patient had tracer-related local or systemic allergic reactions, injection site infection, or any serious adverse events.

## Discussion

In patients with early breast cancer, axillary SLNB is currently the standard method to assess tumor spread to the axilla. In a survey conducted in China, among 110 hospitals performing >200 breast cancer surgeries per year, the majority (69/110, 62.73%) used dye (mainly MB) as the tracer for SLNB; the dual tracer of RI+MB was used only in 16/110 (14.55%) hospitals, probably because of the limited availability of RI tracers (20). Several authors have found ICG to be an excellent tracer in terms of safety, feasibility, and accuracy (21–26). The feasibility of replacing RI with ICG for axillary SLN tracing in breast cancer is currently a hot topic of research.

Recognizing that obesity, age, anatomy, and other factors may affect the success rate of ICG axillary SLN tracing, we adopted a self-controlled protocol to compare the efficacy of different tracers. No significant difference was found between ICG+MB and RI+MB in positivity, axillary SLN detection rate, metastatic axillary SLN detection rate, and number of metastatic axillary SLNs detected. In addition, we compared the effect of the two tracer methods based on the number of lymph nodes. The results showed that ICG+MB was no less than RI+MB, and even higher than RI+MB in terms of detection rate (based on lymph node calculation). These findings are consistent with most previous reports (12, 21, 22, 27, 28). The ICG+MB method detected a significantly larger number of axillary SLNs and may be able to reduce the false-negative rate (29). As far as we know, this is the largest self-controlled study to date comparing the ICG+MB method and the RI+MB method.

The advantages of the RI include longer concentration of radioactivity in the SLN and easier operation, and it allows the surgeon to find the “hot spot” without cutting through the skin, which is not possible with the biological dye method. However, due to radiological contamination of RI and inconvenience of use, it cannot be widely applied in hospitals in China. Near-infrared imaging provides γ-ray tissue penetration, without exposing the patient to radiation. Another advantage is that the cost of ICG is about 21.9% that of RI (8). In our hospital, no matter how many tracers are used, the operation fee is only charged once. In terms of patient costs, patients using the ICG tracer spent less than a third of the cost of RI. The vascular fluorescence imager can provide real-time guidance and greatly reduce the difficulty of surgery; ICG tracing is therefore excellent for training of young breast surgeons (30–32).

In 2020, Goonawardena et al. (23) conducted a systematic review of the application of ICG and RI for breast cancer axillary SLN tracing and reported a detection rate and sensitivity of 81.9%–100% and 65.2%–100%, respectively. They found no significant difference in the detection rate and sensitivity between ICG and RI. In the current study, the detection rate and positivity with ICG were 97.8% and 90.5%, respectively, which is consistent with the results of Goonawardena et al. Although the detection rate with ICG is high, there are disadvantages associated with the use of ICG alone. First, accidental cutting of lymphatic vessels could result in fluorescent pollution of the operative area and make it difficult to identify the truly metastatic axillary lymph nodes and, thereby, also increase procedure time (33). Second, ICG fluorescence penetrance is only about 1 cm, and so metastatic SLN in some deep axillary regions may be missed (5, 34). Using the combination of MB and ICG can solve these problems. Previous studies have shown that the detection time for each axillary SLN is significantly shorter, and the number of axillary SLNs detected significantly more, with ICG+MB than with ICG alone (28, 33).

In this study, the ICG+MB method detected 100% of axillary SLNs. Our result is consistent with a study from Japan, which reported a detection rate >99% (35). In our study, the positivity of ICG+MB was 95.2%, which is similar to the 94.4% reported from our previous study (12). Positivity and the metastatic SLN detection rate were also close to the results of a recent study in China (95.2% vs. 96.6%; 93.7% vs. 94.1%) (36). Interestingly, we found that the number of axillary SLNs detected was higher with ICG+MB than with RI+MB. This has not been reported by other authors. According to previous reports, the mean number of axillary SLNs detected is higher with ICG (1.3–5.4 per patient) than with MB or RI (11, 23, 29, 37–39). Our study also found that a higher number of SLNs were detected by ICG (mean, 4.6 by ICG vs. 3.3 by RI). The explanation may be that the ICG molecules are smaller and so more easily migrate through the lymphatic system; further, fluorescence imaging sensitivity is higher (37, 40). In recent years, surgeons have studied the risk factors for axillary non-sentinel lymph node (nSLN) metastases in patients with axillary SLN metastases. It was found that the number of SLN metastases is an independent risk factor for axillary nSLN metastases (41). Therefore, it is more important to remove multiple SLNs rather than single or two SLNs, especially for surgeons following the Z0011 trial. Direct comparison between ICG+MB method and RI+MB method showed that the efficacy of the ICG+MB method is no less than that of the RI+MB method; this finding is consistent with the results of our previous research (12).

Some scholars consider that neoadjuvant chemotherapy may affect the lymphatic vessels of patients. The neoadjuvant chemotherapy patients enrolled in our study were all patients who had clinically negative axillary lymph nodes examined before surgery and had no downstaging of axillary lymph nodes after neoadjuvant chemotherapy. Moreover, 30 patients with neoadjuvant chemotherapy not only met the requirements of neoadjuvant chemotherapy guidelines but also met the guidelines of sentinel lymph node biopsy (42, 43). Secondly, we further conducted subgroup analysis and compared the positive rate, SLN detection rate, number of SLNs detected, and number of SLN metastases. Our results showed that the introduction of ICG could reduce the impact of neoadjuvant chemotherapy on the number of SLNs detected. In addition, many studies on sentinel lymph node tracers did not include patients with neoadjuvant chemotherapy, which is also one of the characteristics of our study.

In two patients in the present study, the detected axillary SLNs were only ICG positive, but pathological examination confirmed metastases. This suggests that the use of ICG+MB+RI might reduce the false-negative rate with the RI+MB method. However, when using ICG as a tracer, great care must be taken to avoid intraoperative ICG leakage as it may negate the benefits of fluorescence imaging. Exploration should be carried out only after opening the axillary fascia; blind exploration in the fat tissue must be strictly avoided (5).

This study has limitations. First, there was no long-term follow-up, and so data on postoperative recurrence, metastasis, and survival were not available. Second, this was a single-center study with a relatively small sample size; thus, although the data suggest that ICG is a feasible alternative to RI, the findings need to be confirmed in large randomized trials.

## Conclusion

ICG appears to be a reliable, safe, intuitive, and effective tracer for axillary SLNB in patients with breast cancer. Our previous study and the present study indicated that ICG+MB could offer comparable performance compared to RI+MB in SLN mapping. Therefore, ICG may be the preferred method when RI is not available or convenient to use. Multicenter clinical trials are warranted to further verify the current findings.

## Data Availability Statement

The raw data supporting the conclusions of this article will be made available by the authors, without undue reservation.

## Ethics Statement

The studies involving human participants were reviewed and approved by the Medical Ethics Committee of the Southwest Hospital, Third Military Medical University (Army Medical University). The patients/participants provided their written informed consent to participate in this study.

## Author Contributions

XQ and JJ had full access to all the data in the study and take responsibility for the integrity of the data and the accuracy of the data analysis. Study concept and design: YJ, LY, YZ, XQ, JJ. Acquisition, analysis, or interpretation of data: YJ, LY, PT, YY, LF, LC. Drafting of the manuscript: YJ, LY. Critical revision of the manuscript for important intellectual content: XQ, JJ. All authors contributed to the article and approved the submitted version.

## Funding

This study was supported by grants from the Chongqing Science and Health Joint Medical Research Project (No. 2019QNXM003, No. 2020FYYX027) Military Medical Staff Innovation Plan of Southwest Hospital (No. SWH2018BJLC-04), Army Medical University (No. XZ-2019-505-042), and Program of National Key Clinical Specialist Construction (No. 413F1Z113).

## Conflict of Interest

The authors declare that the research was conducted in the absence of any commercial or financial relationships that could be construed as a potential conflict of interest.

## Publisher’s Note

All claims expressed in this article are solely those of the authors and do not necessarily represent those of their affiliated organizations, or those of the publisher, the editors and the reviewers. Any product that may be evaluated in this article, or claim that may be made by its manufacturer, is not guaranteed or endorsed by the publisher.
