# Supplementary material for: A Prospective Self-Controlled Study of Indocyanine Green, Radioisotope, and Methylene Blue for Combined Imaging of Axillary Sentinel Lymph Nodes in Breast Cancer
Source: Front Oncol. 2022 Feb 9;12:803804. doi: 10.3389/fonc.2022.803804 (PMC8863935; doi:10.3389/fonc.2022.803804)
Supplement: Supplementary file 1 [file Table_1.doc]

***Supplemental Material***

Yuting Jin^†^, Long Yuan^†^, Yi Zhang, Peng Tang, Ying Yang, Linjun Fan, Li Chen, Xiaowei Qi*, Jun Jiang*

Department of Breast Surgery, Southwest Hospital, Third Military Medical University (Army Medical University), Chongqing, China

* Correspondence:
Prof. Jun Jiang (E-mail:jcbd@medmail.com.cn) or

Prof. Xiaowei Qi (E-mail:qxw9908@foxmail.com), Department of Breast Surgery, Southwest Hospital, Third Military Medical University, No. 30 Gaotanyan Main Street, Shapingba District, Chongqing, 400038, China.

1. **Supplemental Table 1**

The demographic and clinical characteristics of the 4 patients with no ICG skin lymphatic development and no ICG development in axillary sentinel lymph nodes

|  | Age (years) | BMI (kg/m^2^) | Menopausal status | Neoadjuvant chemotherapy | T stage | Histological type |
| --- | --- | --- | --- | --- | --- | --- |
| case1 | 51 | 23.6 | Premenopausal | No | T2 | Invasive nonspecific cancer |
| case2 | 40 | 28.9 | Premenopausal | No | Tis | In situ |
| case3 | 70 | 21.3 | Postmenopausal | No | T1 | Invasive nonspecific cancer |
| case4 | 71 | 21.2 | Postmenopausal | No | T1 | Invasive nonspecific cancer |

1. **Subgroup analysis of neoadjuvant chemotherapy patients**

**Supplemental Table 2**

Neoadjuvant chemotherapy group vs. non-Neoadjuvant chemotherapy group with triple tracers

| Category | Neoadjuvant chemotherapy | non-Neoadjuvant chemotherapy | P |
| --- | --- | --- | --- |
| SLN number | 4.37±2.47 | 5.22±2.38 | 0.075 |
| Metastatic SLN number | 0.23±0.57 | 0.47±1.10 | 0.247 |
| Positivity | 100%（5/5） | 100%（37/37） | 1.000 |
| SLN detection rate | 100%（30/30） | 100%（152/152） | 1.000 |

**Supplemental Table 3**

*Neoadjuvant chemotherapy vs. non-Neoadjuvant chemotherapy in ICG+MB and RI+MB*

| Category | Neoadjuvant chemotherapy | non-Neoadjuvant chemotherapy | P |
| --- | --- | --- | --- |
| SLN number( ICG+MB) | 4.30±2.45 | 5.13±2.40 | 0.086 |
| SLN number(RI+MB) | 3.07±1.96 | 4.21±2.36 | 0.014 |
| Metastatic SLN number(ICG+MB) | 0.23±0.57 | 0.44±1.08 | 0.307 |
| Metastatic SLN number(RI+MB) | 0.23±0.57 | 0.40±0.93 | 0.341 |
| Positivity(ICG+MB) | 100%（5/5） | 94.6%（35/37） | 1.000 |
| Positivity(RI+MB) | 100%（5/5） | 91.9%（34/37） | 1.000 |
| SLN detection rate(ICG+MB) | 100%（30/30） | 100%（152/152） | 1.000 |
| SLN detection rate(RI+MB) | 100%（30/30） | 98.7%（150/152） | 1.000 |

**Supplemental Table 4**

ICG+MB vs. RI+MB in 30 neoadjuvant chemotherapy patients

| Category | ICG+MB | RI+MB | P |
| --- | --- | --- | --- |
| Positivity | 100%（5/5） | 100%（5/5） | 1.000 |
| SLN detection rate | 100%（30/30） | 100%（30/30） | 1.000 |
| SLN number | 4.30±2.45 | 3.07±1.96 | 0.036 |
| Metastatic SLN number | 0.23±0.57 | 0.23±0.57 |  |

1. **Supplemental Table 5 ICG+MB vs. RI+MB based on SLNs**

|  | **ICG+MB** | **RI+MB** | ***P*** |
| --- | --- | --- | --- |
| Metastatic SLN detection rate（per-SLN basis ） | 74/79 (93.7%) | 68/79 (86.1%） | 0.114 |
| SLN detection rate （per-SLN basis ） | 909/925(98.3%) | 732/925(79.1%) | 0.000 |
